# The effect of peer victimization on adolescents’ revenge: the roles of hostility attribution bias and rumination tendency

**DOI:** 10.3389/fpsyg.2023.1255880

**Published:** 2024-01-11

**Authors:** Xu-Yan Zhao, Shu-Jie Zheng

**Affiliations:** ^1^School of Educational Science, and Institute for Education and Treatment of Problematic Youth, Ludong University, Yantai, China; ^2^Basic Courses Teaching and Research Department, Yingkou Institute of Technology, Yingkou, China

**Keywords:** peer victimization, revenge, hostile attribution bias, rumination tendency, abstract analytic rumination, concrete experiential rumination

## Abstract

Although previous studies revealed that peer victimization was closely related to revenge, mechanisms underlying this association have been unclear. The purpose of this study is to examine the mediating role of hostility attribution bias (HAB) and the moderating role of rumination tendency in the relationship between peer victimization and revenge. The data were collected from 6,622 adolescents. The PROCESS macro of SPSS 26.0 was used to examine the hypotheses. The results show that peer victimization positively associates with revenge. Hostile attribution bias play a partial mediating role between peer victimization and revenge. Both the direct effect of peer victimization on revenge and the first half of the mediating effect of HAB are moderated by rumination tendencies. Specifically, both direct and indirect effects of peer victimization on revenge are stronger in individuals with concrete experiential rumination (CER) tendency than in those with abstract analytic rumination (AAR) tendency.

## 1 Introduction

Peer victimization refers to the experience of individual encounter from peer aggression, including physical aggression, verbal aggression, relationship aggression and property aggression (Mynard and Joseph, 2000; Zhang et al., 2009). Several studies based on large samples have found that peer victimization is common among adolescents. Furthermore, peer victimization has emerged as a global public health issue (Xiao et al., 2023). The victim’s revenge is a deliberate counterattack against the perpetrator and is a type of retaliatory infringement (McCullough, 2017). Revenge is continual and more destructive than other attacks. Revenge against the perpetrator causes a stronger counterattack, which creates a vicious circle and a long-term vendetta (Li and Feng, 2010). Revenge is a global phenomenon and is implicated as a causal factor in many homicides worldwide. Across the United States, revenge is implicated in as many as 61% of school shootings (Jackson et al., 2019). Revenge can drive victim to join terrorist groups and is one of the leading causes of regional conflicts around the world (Chen et al., 2016). To prevent adolescents from breaking the law for revenge, it is necessary to explore the formation mechanism of revenge. This will help to prevent adolescents who have experienced peer victimization changing from “victims” to “perpetrators.”

Previous studies have explored the relationship between peer victimization and general aggressive behavior of adolescents from different perspectives, such as adolescents’ negative companionship (Chen, 2019), unmet psychological needs (Wang et al., 2020), and lack of parental supervision (Ye et al., 2018). However, revenge is a special type of reactive aggression, which is usually an individual’s deliberate counterattack against an perpetrators. Revenge is planned and delayed. Cognitive factors play a central role in maintaining revenge and are stable across time and context (Rowell Huesmann, 1988; Crick and Dodge, 1994). Therefore, this study analyzes the path and conditions of the relationship between peer victimization and adolescent revenge from a cognitive perspective. The results of the study will contribute to the prevention and intervention of adolescent revenge.

### 1.1 Relationship between peer victimization and adolescent revenge

Revenge is a means of self-protection chosen by an individual who has been victimized, as well as a compensatory mechanism in the hope of making up for the loss of dignity as a result of the violation (Li and Feng, 2010). A recent meta-analytic study found that peer victimization is not only strongly associated with perpetration, but it is also a risk factor for future violation (Walters, 2020). Relevant studies have shown that in order to maintain dignity, regain social status and satisfy certain psychological needs, the victim tries to reduce the negative emotions associated with peer victimization by retaliating against the bully (Ye et al., 2018). When individuals feel humiliated, revenge is seen as a way to restore dignity and regain control of the situation (Fitness, 2001).

### 1.2 The mediating role of hostile attribution bias

Hostile attribution bias (HAB) refers to the cognitive propensity of individuals to view the behavioral motives of others in ambiguous circumstances as intentional to harm themselves (Dodge, 2006). Previous research has shown that hostile attribution bias predicts reactive aggression and is an important cognitive factor in the formation and development of reactive aggression (Crick and Dodge, 1996). However, there is a lack of research specifically on the link between HAB and revenge.

According to social information processing theory, when individuals process new information, they spontaneously interpret it using experiences related to it in memory, providing *a priori* experiences, and ndividuals’ interpretations of social situations affect their subsequent behavior (Crick and Dodge, 1994; Xiang et al., 2022). Empirical research supports the notion that peer victimization causes individuals to construct schemas of distrust, and such distrustful schemas can cause adolescents to interpret their peers’ intentions hostilely in ambiguously provocative situations (Li et al., 2021). After perceiving provocation, adolescents interprete the provocative behavior as being hostile, which is significantly associated with seeking revenge (Smalley and Banerjee, 2014). The tracking study shows that peer victimization changes preadolescent children’s psychological structures, leading to the formation of HBA, which leads to future aggressive behavior (Yao and Enright, 2021). Therefore, Adolescents who suffer from peer victimization perceive harm in interpersonal communication, which provides prior experience for their judgment of interpersonal events. These prior experiences can lead to hostile attribution bias in adolescents, which in turn prompts them to resort to revenge to protect themselves.

### 1.3 The moderating role of rumination tendency

Cognitive strategies are a way for individuals to view and understand emotional events and can significantly affect their emotional and behavioral responses (Chawla and Ostafin, 2007; Zhang, 2020). Studies have confirmed that adolescents’ coping strategies for emotional events gradually shift from external behavioral orientation to internal cognitive orientation (Luo et al., 2010; Xu, 2014). As a cognitive coping strategy, rumination plays an important role in adolescents’ cognitive evaluation and behavioral response to peer victimization. However, rumination is largely recognized as maladaptive in existing research, but such studies have been conducted primarily in Western contexts (Choi and Miyamoto, 2023). A growing body of cross-cultural research suggests that there are cultural differences in the frequency of rumination and its relationship to psychological outcomes (Chang et al., 2010; Kwon et al., 2013). The maladaptive effects of rumination may be weaker in Eastern than in Western cultural contexts (Choi and Miyamoto, 2023). Based on this, this study explores the role of rumination tendency in the relationship between peer victimization and adolescent revenge in the Chinese cultural context.

Rumination is the repeated thinking about negative events and their causes and consequences. Rumination can be divided into abstract analytic rumination (AAR) and concrete experiential rumination (CER) based on the processing method (Denson, 2013). AAR is the repeated thinking about the possible causes and consequences of the negative events (i.e., a“why”focus) experience of peer victimization. CER is the individual’s repeated thinking about the process, situation and details of the negative events (i.e., a “what” focus). AAR induces an abstract processing style by contrast, CER induces a concrete processing style (Watkins, 2004). According to construal level theory, people have different levels of abstraction in their representations of events. A high level of abstraction means a high-level construal, and a low level of abstraction means a low-level construal (Trope and Liberman, 2003; Zhang et al., 2018). People with high-level construals pay more attention to the essence of the event, tend to view things from a macro and long-term perspective, and think about the overall situation, which is conducive to individual self-control. People with low-level construals focus on the details of the event and tend to see things from a micro and short-term perspective for the sake of immediate interests, which leads to a failure of self-control (Chen, 2016). Several studies have shown that high-level construals promote self-control and thus make individuals focus more on the long-term benefits of events rather than the immediate concrete outcomes; these individuals thus exhibit more self-control behaviors (Huang et al., 2015; Zhang et al., 2018). The research results of the adolescent group suggest that the construal level is significantly positively correlated with the control system of self-control and is significantly negatively correlated with the impulse system of self-control (Wu et al., 2022). Given the above findings, adolescents who encounter peer victimization and individuals with an AAR tendency may have lower levels of revenge than those with a CER tendency.

Studies in psychotherapy find that after a negative event, writing down one’s feelings, exploring the possible causes of the event, and analyzing the influence of the event can promote the process of psychological recovery and that AAR is similar to this process (Guo and Wu, 2011a,b). Chinese culture also involves idioms that are similar to AAR, such as “Every day I do self-inspection on three aspects.” Reflection and review are regarded as activities that promote self-growth in Chinese culture. Therefore, AAR has the potential to promote the cognitive and social adaptation of individuals. AAR is likely to enable adolescents to recognize their own shortcomings and areas for improvement in peer victimization as well as the possible serious consequences of revenge, which in turn leads to multiple interpretations of the provocateur’s intentions. Therefore, the level of HAB may be lower in individuals with AAR than in those with CER.

### 1.4 The present study

**Figure 1:**
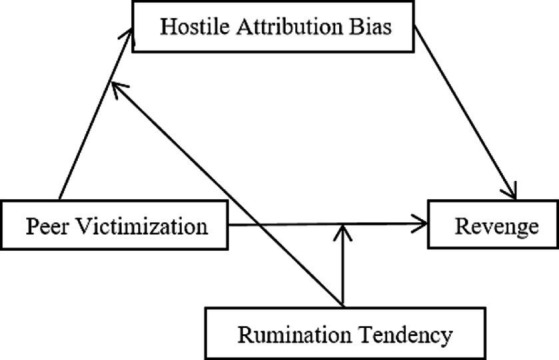
The proposed moderated mediation model.

It is important to note that the rumination process in daily life is uncontrollable and involuntary. However, most existing studies on rumination are based on experimental studies of the rumination-induction paradigm. This may make the ecological validity of the rumination operation in the experimental study insufficient. Moreover, existing research has mostly used trait rumination questionnaires and the respondents have mostly been college students; youth groups have rarely been involved. This study used the Adolescent Peer Victimization Rumination Questionnaire to investigate the role of rumination tendency between peer victimization and adolescent revenge. Based on social information processing theory (Crick and Dodge, 1994) and construal level theory (Trope and Liberman, 2003), the present study regards hostile attribution bias as the mediating factor in the relationship between peer victimization and revenge and further explores the moderating role of rumination tendencies on this process. The hypothesis model is shown in Figure 1.

According to the previous research, First, we expected that higher levels of peer victimization would report higher levels of revenge behaviors (Hypothesis 1). Second, we hypothesized that Hostile attribution bias would mediate the relationship between peer victimization and adolescent revenge (Hypothesis 2). Third, we hypothesized that Rumination tendency would moderate the relationship between peer victimization and adolescent revenge. Specifically, compared with individuals with an AAR tendency, peer victimization would have a larger role on adolescent revenge for those with a CER tendency (Hypothesis 3). Finally, we hypothesized that rumination tendency would moderate the relationship between peer victimization and hostile attribution bias. Specifically, compared with individuals with an AAR tendency, peer victimization would have a larger role on hostile attribution bias for those with a CER tendency (Hypothesis 4).

## 2 Materials and methods

### 2.1 Participants and procedure

The sample for this study was drawn from October to December 2022, during the COVID-19 outbreak, when students began to attend online classes at home. The survey research complied with the relevant normative requirements of the Research Ethics Committee of Ludong University. Online questionnaires were used to obtain data. The link to the online questionnaire was delivered to participants via classroom instructors, and participants completed it independently. The purpose of the survey was explained to the participants on the first page of the questionnaire, and the respondents were ensured that personal information would be kept strictly confidential. This study got informed consent from the participants and their fathers or mothers, and the participants volunteered to participate anonymously.

This study used cluster sampling to select 35 junior high schools and 8 senior high schools from five provinces: Liaoning, Inner Mongolia, Jilin, Guizhou and Sichuan. A total of 18,914 students participated in the survey. We excluded invalid questionnaires based on the following criteria: an excessively short completion time, errors in the answer to the attention point monitoring item, and an answer of less than 7 points out of a possible 10 on a self-rated seriousness question. Referring to the screening method of existing studies (Zhang et al., 2009; Guo et al., 2017), students with a score of 1 (never) on each factor of peer victimization were categorized as “non-victims,” those with a score of more than 1 but less than 3 (sometimes) were categorized as “seldom victims,” and those with a score of 3 or more were categorized as “frequent victims.” Among the valid samples, a total of 6,622 students have experienced peer victimization (including “seldom victims” and “frequently victims”), which is 5,198 junior high school students (grades 7–9) and 1,424 senior high school students (grades 10–12). 3,098 (46.78%) were boys and 3,524 (53.22%) were girls, with an age range for junior high school students of 13–16 years and an age range for senior high school students of 16–18 years.

### 2.2 Measures

#### 2.2.1 Multidimensional peer victimization scale

The revised version of the Multidimensional Peer Victimization Scale (Guo et al., 2017) was used to measure participants’ experiences of peer victimization. It contains four dimensions: physical victimization (3 items), verbal victimization (5 items), relational victimization (7 items) and property victimization (3 items). Participants used a 4-point Likert scale (1 = never happened to 4 = often happened). Cronbach’s alpha was 0.92 in this study. Confirmatory factor analysis showed that the construct validity of the scale was good (GFI = 0.94, CFI = 0.94, AGFI =0.92, RMSEA = 0.063).

#### 2.2.2 Revenge scale

The revenge subscale of the Revised Transgression-Related Interpersonal Motivations Inventory (Chen et al., 2006) was used. Participants were required to evaluate their revenge level on 5 items (e.g.,“I will make him/her pay.”) using a 5-point Likert scale (1 = strongly disagree to 5 = strongly agree). The Chinese version of this subscale showed good applicability. Cronbach’s alpha was 0.90 in this study. Confirmatory factor analysis showed that the construct validity of the scale was good (GFI = 0.99, CFI = 0.99, AGFI = 0.98, RMSEA = 0.056).

#### 2.2.3 Word sentence association paradigm-hostility

The revised version of the Word Sentence Association Paradigm-Hostility (Zhang, 2019) was used to measure participants’ hostile attribution bias. It contains 11 am biguous circumstances, and each circumstances is followed by a word related to hostility (e.g., “your friend did not respond to what you said: ignored”). Participants used a 5-point Likert scale (1 = strongly disagree to 5 = strongly agree). The questionnaire had good internal consistency, test–retest reliability and validity. Cronbach’s alpha was 0.95 in this study. Confirmatory factor analysis showed that the construct validity of the scale was good (GFI = 0.96, CFI = 0.98, AGFI =0.93, RMSEA = 0.075).

#### 2.2.4 Peer victimization rumination questionnaire

We measured the rumination tendency of adolescents after peer victimization with self-administered Peer Aggression Rumination Questionnaire. The 13-item questionnaire includes two dimensions: abstract analytic rumination (AAR,7 items, e.g., “I think over and over again, trying to find out why he/she (they) hurt me”) and concrete experiential rumination (CER, 6 items, e.g., “I cannot help thinking back to the details of my being bullied”). Responses are rated on a 5-point Likert scale (1 = never to 5 = always). Referring to the calculation method of Simple Coping Style Questionnaire (Dai, 2015), Rumination tendency = Z-score of CER - Z-score of AAR. Rumination tendency value greater than 0 suggests that the participant primarily uses CER. Rumination tendency value less than 0 suggests that participant mainly used AAR. Cronbach’s alpha was 0.92 in this study. Confirmatory factor analysis showed that the construct validity of the scale was good (GFI = 0.96, CFI = 0.98, AGFI = 0.95, RMSEA = 0.060).

### 2.3 Data analysis

The effective data collected were analyzed by SPSS 26.0 software. Firstly, the Harman univariate test was used to test the existence of common method bias. Descriptive statistics and correlation analysis were conducted for the 4 variables to determine the relationship between the variables. In addition, collinearity diagnosis was performed to confirm the existence of multiple collinearities among variables. Secondly, model 8 in PROCESS version 3.5 of SPSS 26.0 macro program was used to test the moderating effect of rumination tendency.

## 3 Results

### 3.1 Common method bias test, multicollinearity diagnosis and correlation analyses of variables

On the one hand, by using an anonymous measuring approach, the common method deviation was reduced. The common method deviation, on the other hand, was regulated from the standpoint of statistical control following data collection by examining the exploratory factor analysis results. We used Harman single factor test to examine common method deviation. The results showed that KMO and Bartlett Test of Sphericity results was 0.966 (*p* < 0.001), indicating that it was suitable for the factor analysis. There were 6 factors with eigenvalues greater than 1, and the explained variance of the first factor was 32.21% (less than 40%; Deng et al., 2018), suggesting that common method deviation was not a serious threat in this study.

Furthermore, the variance inflation factor (VIF) was used to establish the occurrence of multicollinearity amongst variables (Yang et al., 2012). The findings reveal that the VIF values ranged from 1.30 to 1.81, significantly less than the critical value of 10, while tolerance values ranged from 0.55 to 0.72, which were greater than 0.1 (Zhao et al., 2023) Therefore, there was no multicollinearity problem in the mode.

According to the pearson correlation analysis, the primary variables were significantly correlated with each other (see Table 1). Specifically, pairwise significant positive correlations were found between peer victimization, revenge, hostility attribution bias and rumination tendency. The demographic variables of gender, grade, school location, boarding situation, and left-behind status were significantly correlated with the main variables, and they were included in the model as control variables in the subsequent moderated mediation analysis.

**Table 1:** Descriptive statistics and correlations among all variables.

Variables	*M*	*SD*	1	2	3	4
1. Peer victimization	1.56	0.53	1			
2. Revenge	2.77	1.02	0.44**	1		
3. Hostile attribution bias	2.10	1.00	0.53**	0.57**	1	
4. Rumination tendency	–	–	0.30**	0.52**	0.55**	1
5. Gender	–	–	0.04**	0.03*	0.02	0.01
6. Grade	–	–	0.05**	0.11**	0.09**	0.02
7. School location	–	–	−0.04**	0.02	0	0
8. Boarding	–	–	−0.01	−0.03*	−0.02	0.01
9.Left behind status	–	–	−0.02*	−0.02	−0.03*	0

*p < 0.05, **p < 0.01.

### 3.2 Moderated mediation analysis

Every variable was standardized. Model 4 of the PROCESS macro was applied to assess the mediation effect of hostile attribution bias. Gender, grade, school location, boarding and left-behind status were control variables. Peer victimization significantly positively predicted revenge (*β* = 0.45, *p* < 0.001). Peer victimization significantly positively predicted hostile attribution bias (*β* = 0.53, *p* < 0.001). After incorporating hostile attribution bias into the regression equation, hostile attribution bias significantly positively predicts revenge. Peer victimization can still significantly positively predict revenge (*β* = 0.20, *p* < 0.001). Bootstrap analysis indicated that the direct effect of peer victimization on revenge was significant [effect size = 0.20, Boot SE = 0.01; 95% CI = (0.18, 0.22)], and the mediating effect of hostile attribution bias was significant [effect size = 0.24, Boot SE = 0.01; 95% CI = (0.23, 0.26)]. Therefore peer victimization can not only directly predict revenge but can also predict revenge through the mediating role of hostile attribution bias. The direct effect and mediating effect accounted for 45.45 and 54.55% of the total effect, respectively.

**Figure 2:**
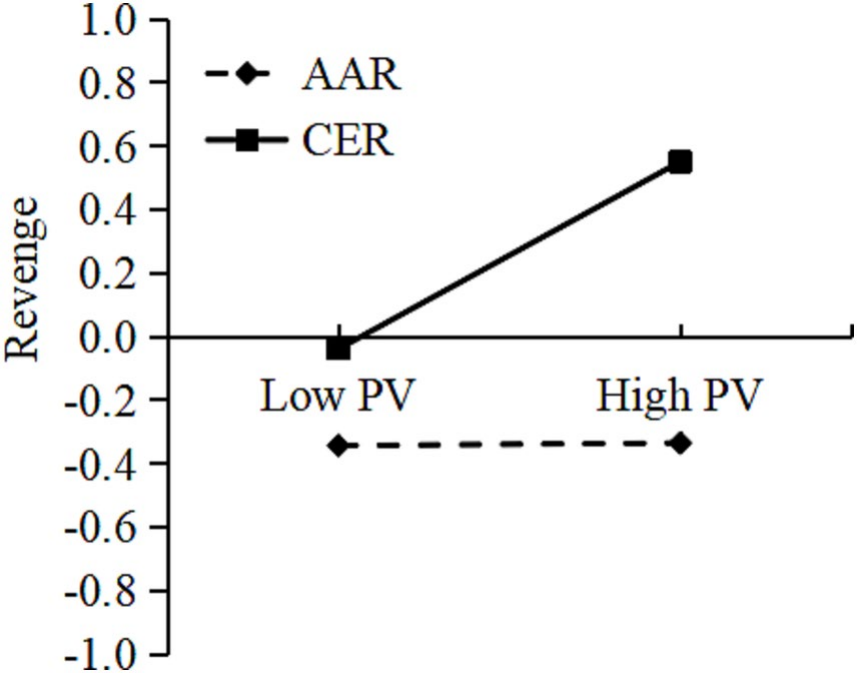
Rumination tendency as a moderator in the relationship between peer victimization and revenge. Low pv, Low Peer Victimization; High pv, High Peer Victimization.

**Figure 3:**
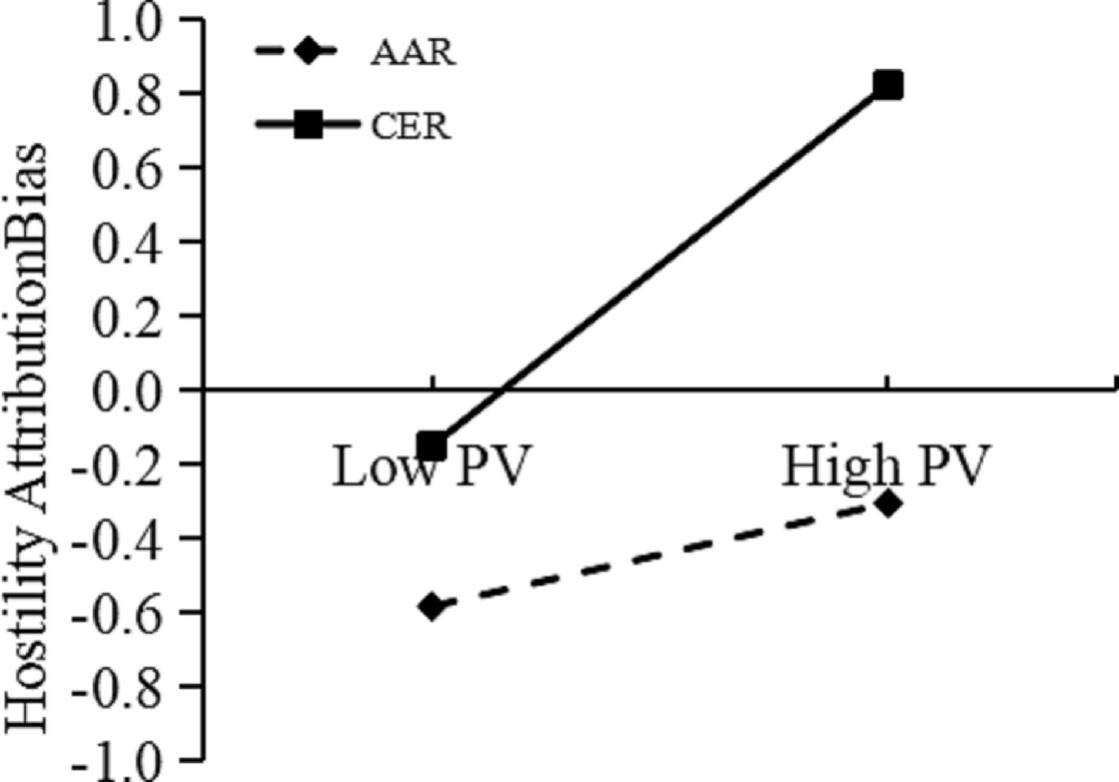
Rumination tendency as a moderator in the relationship between peer victimization and hostile attribution bias. Low pv, Low Peer Victimization; High pv, High Peer Victimization.

Furthermore, we examined the moderating effect of rumination tendency using Model 8 of the PROCESS macro. The results showed that (see Table 2) the interaction between peer victimization and rumination tendency significantly predicted hostile attribution bias [*β* = 0.14, *p* < 0.001, 95% bootstrap CI (0.13, 0.16)], and also significantly predicted revenge [*β* = 0.12, *p* < 0.001, 95% bootstrap CI (0.11, 0.13)]. Therefore, we came to the conclusion that rumination tendencies significantly moderated both the intermediary first half path and the direct path. In order to more intuitively illustrate the moderating effect of rumination tendency, the study defined the mean of the rumination tendency variable score, plus one standard deviation of the data as CER support data, and the sample mean minus one standard deviation of the data as AAR support data. As a result, a simple slope effect diagram with different degrees of rumination tendency was displayed in Figures 2, 3. Figure 2 shows that for participants who tended to engage in CER, peer victimization significantly predicted revenge, simple slope = 0.30, *t* = 24.31, *p* < 0.05. For participants who tended to engage in AAR, peer victimization had no significant effect on revenge, simple slope = 0.01, *t* = 0.48, *p* > 0.05. This finding demonstrated that the higher the individual’s proclivity toward CER, the greater the positive predictive effect of peer victimization on revenge. Figure 3 shows that for participants who tended toward CER, peer victimization had a significant positive predictive effect on hostile attribution bias, simple slope = 0.49, *t* = 49.32, *p* < 0.001; for participants who tended toward AAR, although peer victimization also had a positive predictive effect on HAB, its predictive effect was weaker, simple slope = 0.14, *t* = 9.85, *p* < 0.001. This finding indicated that the individuals tended toward CER, the higher the predictive effect of peer victimization on hostile attribution bias. Furthermore, at the three levels of rumination tendency, as the rumination tendency changes from AAR to CER, peer victimization is more likely to induce revenge by increasing hostile attribution bias.

**Table 2:** The moderated mediation model.

Regression equation		Overall fit index			Significance of regression coefficient			
outcome	Predictors	*R*	*R* ^2^	*F*	*β*	LLCI	ULCI	*t*
HAB	Gender	0.70	0.50	812.06***	0.01	−0.03	0.04	0.49
	Grade				0.05	0.03	0.06	7.27***
	School Location				0.01	−0.03	0.05	0.33
	Boarding				0.02	−0.03	0.07	0.86
	Left behind				−0.02	−0.06	0.02	−0.96
	PV				0.31	0.29	0.33	31.85***
	RT				0.32	0.31	0.34	42.07***
	PV × RT				0.14	0.13	0.16	24.11***
Revenge	Gender	0.67	0.45	589.60***	0.03	−0.01	0.07	1.57
	Grade				0.05	0.04	0.06	7.31***
	School Location				0.04	−0.01	0.08	1.67
	Boarding				0.01	−0.04	0.06	0.44
	Left behind				0.01	−0.03	0.04	0.25
	PV				0.15	0.13	0.17	13.62***
	HAB				0.23	0.20	0.25	17.63***
	RT				0.25	0.23	0.27	27.26***
	PV × RT				0.12	0.11	0.13	18.20***

All variables in the model were standardized and brought into the regression equation.

PV, Peer Victimization; HAB, Hostile Attribution Bias; RT, Rumination Tendency.

*p < 0.05; **p < 0.01; ***p < 0.001.

## 4 Discussion

This study explored the relationship and internal mechanism of peer victimization on adolescents’ revenge. We analyzed the mediating role of hostility attribution bias and the moderating role of rumination tendency. This study found that peer victimization significantly positively predicted revenge. The stronger the degree of peer victimization, the higher the possibility of revenge among adolescents, which supported research Hypothesis 1. The result aligned with social information processing theory (Crick and Dodge, 1994). That is, people encode social cues and deduce others’ behavioral intentions based on previous experience, beliefs, and situations, which affect the response to current events and lead to subsequent behavior.

Consistent with the conclusions of previous studies, frequent peer victimization aggravates individuals’ subjective perception of peer hostility. Individuals can quickly identify the hostile behavior intentions of peers due to their previous experience of being abused; thus, more aggressive behaviors are used to cope with conflicts (Xiao et al., 2023). At the same time, this may amplify the hostility of individual subjective perceptions of peer behavior intentions (Schacter and Juvonen, 2015). Adolescents may believe that their peers are deliberately targeting them, prompting them to take more revenge to resolve peer conflicts. This makes them change from the “victim” to the “perpetrator.”

This study found that hostile attribution bias played a mediating role between peer victimization and adolescent revenge, which supported research Hypothesis 2. The conclusion of this study supports the social information processing model (Crick and Dodge, 1994). Individuals’ social experiences affect their cognition and play a role in behavioral responses in later social interactions. The conclusions of the study verify the research inference of Graham and Juvonen (1998) that hostile attribution bias is related to the revenge behavior of middle school students, which may be derived from previous experiences of being hurt by peers. As suggested by the results of several existing experimental studies, hostile attribution bias not only leads to aggression but also maintains aggressive behavior patterns (Verhoef et al., 2019). Actual peer victimization activates a hostile cognitive bias, which in turn predicts subsequent aggressive responses (DeWall et al., 2009; Reijntjes et al., 2011). That is, peer victimization changes the way youth attend to social cues, by increasing their hypervigilance to hostile cues (Reijntjes et al., 2011). This helps us to better understand the recurrence and vicious cycle of revenge. In summary, peer victimization is an important risk factor that affects individual cognitive development, which makes adolescents tend to have negative views of interpersonal events. Adolescents’ attributional bias toward an aggressor’s behavioral intentions is a powerful predictor of whether the adolescent will attempt revenge. Therefore, It is of great practical significance to explore the influence of peer victimization on revenge from the perspective of adolescent cognition to prevent the revenge of adolescents who suffered from peer victimization.

The present study found that the effects of peer victimization on adolescents’ hostile attribution bias and revenge were moderated by rumination tendencies. First, rumination tendency moderated the direct path of peer victimization on revenge, supporting research Hypothesis 3. The positive predictive effect of peer victimization on revenge was stronger for adolescents who tended toward CER than for those who tended toward AAR. Consistent with past research that rumination play an essential role in which victimization and life stress forecasts externalizing issues such as bullying perpetration (Malamut and Salmivalli, 2021) and delinquent and aggressive conduct (LeMoult et al., 2019). Second, rumination tendency moderated the predictive effect of peer victimization on hostile attribution bias, supporting research Hypothesis 4. Peer victimization had a stronger positive predictive effect on hostile attribution bias for individuals who tended toward CER than for adolescents who tended toward AAR. This supports the findings of Pedersen et al. (2011) that provocation-focused rumination (i.e., dwelling on a specifc grievance or occurrence) predicted aggressive cognition. However our conclusions are contrary to the findings of studies on depression rumination. Research on depression rumination suggests that CER contributes to problem solving compared to AAR (Watkins and Moulds, 2005). AAR can cause individuals to maintain or enhance anger (Denson et al., 2012), produce negative overgeneralizations (Watkins, 2008). We found that CER and AAR, in existing studies, focus on negative emotions (e.g., depression or angry) or the self (e.g., why am I always anxious), but AAR may be more conducive to individual to coping negative events objectively and rationally when the content of the rumination focused on a specific event experienced rather than on negative emotions or the self in the context of Chinese culture. The theoretical model of triggered displaced aggression (Miller et al., 2003) provides some explanation for the link between rumination tendencies and revenge. This theory suggests that infringement elicits negative emotions in the victim, which in turn activates related cognitive and motivational structural nodes within the same associative network. The association of these nodes makes adolescents perceive and respond to interpersonal harm and forms memory imprints in a network of associations. Thus, the more individuals tend to engage in CER, the more they repeatedly experience emotional feelings when they are hurt, maintaining the infringement network that is activated by the initial provocation. Furthermore, the cognitive (hostile attribution bias) and motivational (revenge motivation) nodes associated with the peer victimization event may be reactivated (Zhang et al., 2015; Ruddle et al., 2017). In contrast, adolescents who tend toward AAR conduct an in-depth rational analysis of the possible causes of peer victimization and the possible consequences of revenge, and they have multiple interpretations of their peers’ intentions. This helps the victim recall the experience of being bullied with calm cognition and examine peer victimization events without reactivating negative emotions. Supported by the research of Ding and Qian (2020), individuals who tend toward AAR can better deal with stimulating events and view problems more objectively and rationally. Current research extends past research to prove that rumination tendencies play a moderating role between peer victimization and revenge. We attempted to categorize negative experience rumination from the perspective of cognitive processing mode. The results of the study found that in the context of Chinese culture, which advocates introspection, AAR may play a constructive role in responsing negative events compared with CER.

## 5 Contribution and implications

In the literature on peer victimization, the victim’s retaliation has always been a neglected area. Although studies have confirmed the strong association between peer victimization and revenge, the mechanisms by which peer victimization affects revenge are unclear. A review of existing research indicates that most current studies use the concept of general aggression. Few studies have distinguished between subtypes of proactive and reactive aggression. Revenge, as a sub-type of reactive aggression, is a delayed reactive aggression, unlike immediate reactive aggression. As many SIP models suggest, in-the-moment behaviors are often not carefully considered, but are more automatic and reflexive. Reflexive socialcognitive processes are automatic, fast, and unconscious whereas reflective social-cognitive processes are relatively conscious and more deliberate (Evers et al., 2014). Some Avengers will wait months or even years to carry out their revenge. What happens in people’s minds when they make these decisions? People’s appraisal of a transgression is a strong predictor of whether they will take revenge (Jackson et al., 2019). Mind perception is a key factor in these appraisals (Young et al., 2011). However, few studies have explored the cognitive mechanisms of revenge. The present study concentrated on the above issues and explored the relationships among peer victimization, hostile attribution bias, rumination tendencies and revenge from a cognitive perspective. It enriched the research category of reactive aggression. It also provided empirical support for scientific prevention of revenge. In addition, we demonstrated the moderating role of rumination tendency and made new findings on the effects of abstract analytic rumination. The current study, to our knowledge, is the first to classify the victimized experience rumination based on a cognitive processing model, and then to explore the moderating role of peer victimization rumination tendencies on revenge and hostile attribution bias. Our findings emphasize the need to dialectically view the role of different rumination tendencies in different cultural contexts. Rumination does not always lead to same outcomes, and interventions for maladaptive coping strategies in adolescents after peer victimization should primarily target concrete experiential rumination (CER) rather than abstract analytical rumination (AAR).

## Data availability statement

The datasets presented in this study can be found in online repositories. The names of the repository/repositories and accession number(s) can be found in the article/supplementary material.

## Ethics statement

The studies involving human participants were reviewed and approved by Ludong University Ethics Committee. Written informed consent to participate in this study was provided by the participants’ legal guardian/next of kin.

## Author contributions

X-YZ: Conceptualization, Investigation, Software, Writing – original draft, Writing – review & editing. S-JZ: Conceptualization, Data curation, Methodology, Writing – review & editing.
